# Real-world use of natalizumab in Austria: data from the Austrian Multiple Sclerosis Treatment Registry (AMSTR)

**DOI:** 10.1007/s00415-023-11686-2

**Published:** 2023-04-19

**Authors:** Tobias Monschein, Sarinah Dekany, Tobias Zrzavy, Markus Ponleitner, Patrick Altmann, Gabriel Bsteh, Barbara Kornek, Paulus Rommer, Christian Enzinger, Franziska Di Pauli, Jörg Kraus, Thomas Berger, Fritz Leutmezer, Michael Guger, the Austrian MS Treatment Registry (AMSTR)

**Affiliations:** 1 Department of Neurology, Medical University of Vienna, Waehringer Guertel 18-20, 1090 Vienna, Austria; 2 Department of Neurology, Karl-Franzens University, Graz, Austria; 3 Department of Neurology, Medical University of Innsbruck, Innsbruck, Austria; 4 Department of Neurology, Medical Faculty, Heinrich Heine University Düsseldorf, Düsseldorf, Germany; 5 Department of Laboratory Medicine, Paracelsus Medical University and Salzburger Landeskliniken, Salzburg, Austria; 6 Clinic for Neurology 2, Kepler University Clinic, Linz, Austria

**Keywords:** Multiple sclerosis, Natalizumab, Effectiveness, Safety, PML

## Abstract

**Introduction:**

With the approval of natalizumab in Europe in 2006, the Austrian Multiple Sclerosis Therapy Registry (AMSTR) was established. Here, we present data from this registry about effectiveness and safety of natalizumab in patients treated up to 14 years.

**Patients/methods:**

Data retrieved from the AMSTR contained baseline characteristics and biannual documentation of annualised relapse rate (ARR) and Expanded Disability Status Scale (EDSS) score as well as adverse events and reasons for discontinuation on follow-up visits.

**Results:**

A total of 1596 natalizumab patients (71% women, *n = *1133) were included in the analysis and the observed treatment duration ranged from 0 to 164 months (13.6 years). The mean ARR was 2.0 (SD = 1.13) at baseline, decreasing to 0.16 after 1 year and 0.01 after 10 years. A total of 325 patients (21.6%) converted to secondary progressive multiple sclerosis (SPMS) during the observational period. Of 1502 patients, 1297 (86.4%) reported no adverse events (AE) during follow-up visits. The most common reported AEs were infections and infusion-related reactions. John Cunningham virus (JCV) seropositivity was the most common specified reason for treatment discontinuation (53.7%, *n = *607). There were five confirmed cases of Progressive Multifocal Leukoencephalopathy (PML) with 1 death.

**Conclusion:**

The effectiveness of natalizumab in patients with active relapsing–remitting multiple sclerosis (RRMS) could be confirmed in our real-world cohort even after follow-up of up to 14 years, though after year 10, there were less than 100 remaining patients. A low number of AE were reported in this nationwide registry study, establishing Natalizumab’s favourable safety profile during long-term use.

## Introduction

Since the approval of interferon-beta in Europe in 1995, the therapeutic landscape in multiple sclerosis has evolved significantly [1]. Since then, over a dozen of disease-modifying therapies (DMTs) have been approved, leading to the dilemma of selecting the most appropriate DMT for a given patient. Since pivotal phase 3 trials are usually limited to 2 years, knowledge about long-term safety (and effectiveness) is limited for new DMTs. Furthermore, health authorities ask for real-world effectiveness data to justify the partly enormous costs of DMTs. In Austria, a country with over 13,000 MS patients, the Austrian Multiple Sclerosis Therapy Registry (AMSTR) was therefore established in 2006 [2, 3].

Natalizumab (NTZ), a monoclonal antibody directed against the adhesion molecule α4ß1-integrin (VLA-4), remains one of the most effective immunomodulatory therapies, with progressive multifocal leukoencephalopathy (PML) being the most severe adverse event [4].

The excellent efficacy of NTZ in highly active RRMS has already been demonstrated in pivotal phase III trials, with a 68% reduction in annualized relapse rate (ARR) and a 42% risk reduction for sustained disability progression [5, 6].

The Tysabri Observational Programme (TOP) strengthens these data with a 92.5% reduction in ARR over 10 years in a real-world treatment setting [7]. Furthermore, the TOP 10-year data confirm the favourable long-term safety profile of NTZ. Infections (4.1%) and immune system disorders (1.5%) were the most common side effects, and in particular, the PML rate was 0.9%.

The aim of this paper is therefore to characterize a cohort of NTZ-treated patients from the AMSTR and to analyse follow-up data on effectiveness and safety with a follow up period of up to 14 years.

## Methods

In Austria, a dense network of certified MS centres, either hospital-based or office-based, provide high-quality patient care and support, ensuring appropriate use and monitoring of DMT treatment. The Austrian MS Treatment Registry (AMSTR) was established in 2006 to acquire data on effectiveness and safety for approved DMTs in a real-world setting, and thereby to monitor emerging long-term effects and risks. Moreover, data entry into the AMSTR is necessary to comply with reimbursement regulations of the Austrian sick funds. The AMSTR is a secure web-based platform, which requires immediate online documentation during patient visits from treating neurologists in all Austrian MS centres.

The initial entry of baseline data into the AMTR captures: MS onset and duration, relapses in the prior 12 months (ARR), expanded disability status scale (EDSS), MRI activity (3 variables: ≥ 9 T2-hyperintense lesions, ≥ 1 contrast-enhancing lesion [CEL], dynamic of lesion load as compared to a previous scan) and previous DMTs. The follow-up data include: relapses, EDSS, adverse events (AE), change or discontinuation of treatment, anti-JCV antibodies (STRATIFY test), neutralizing anti-NTZ antibodies status and is required to be captured every 3–6 months. In the case of treatment withdrawal, additionally, data regarding the reason for discontinuation are assessed.

All patients were categorized into two groups according to EMA indications for NTZ:

Indication A (*escalation strategy*): ≥ 1 relapse despite DMT within the last 12 months and ≥ 9 T2 lesions on a recent (not older than 3 months before treatment start) MRI.

Indication B (*early intensive strategy*): ≥ 2 severe relapses without prior DMT and ≥ 1 gadolinium-enhancing lesion or an increase in T2 lesion burden on a recent MRI compared with a previous scan.

EDSS progression was defined as worsening in the EDSS scale of 0.5 points over two consecutive follow-up visits (equals a median time of 6.2 months). Hence, approximately to the diagnostic criteria of Lorscheider, patients with an EDSS progression of at least 0.5 over at least 6 months without the presence of a relapse in the same time period were therefore rated as secondary-progressive multiple sclerosis (SPMS) [8].

To improve the quality and reliability of the documented data, an independent data monitoring board was established [9]. Since NTZ prescribing and administration is restricted to specialized MS centres, the AMSTR inherently covers NTZ use in Austria. The respective data sets were obtained anonymously and exported from the registry. The study was approved by the Ethics Committee of the Medical University of Vienna (EC number 1448/2020).

### Statistics

Descriptive analysis was conducted using IBM SPSS (SPSS Inc. Version 25.0 and 26.0, Chicago, IL, USA). Categorical variables were expressed in frequencies and percentages, continuous variables were described as mean and standard deviation or median and range in accordance with the presence/absence of normal distribution as indicated by Kolmogorov–Smirnov tests.

Univariate correlations were performed by Pearson or Spearman.

Time to on-treatment relapse as well as treatment discontinuation were assessed by survival analyses in terms of Kaplan–Meier curves and log-rank test.

The following cases were censored: no relapse until data retrieval or until drop-out; no drop-out until data retrieval, lost-to-follow-up, drop-out due to relocation, drop-out due to withdrawal of consent, drop-out due to treatment being moved to a non-AMSTR clinic and death.

Group differences were illustrated applying chi^2^ tests for categorical variables, and independent *t*-test/Mann–Whitney-U tests for continuous variables with/without normal distribution.

A two-sided *p* value of 0.05 was considered the level of significance.

## Results

Baseline data in the AMSTR were available for 1602 patients. Six cases were excluded from the baseline analyses due to insufficient data (unavailable date of the first NTZ administration).

**Fig. 1:**
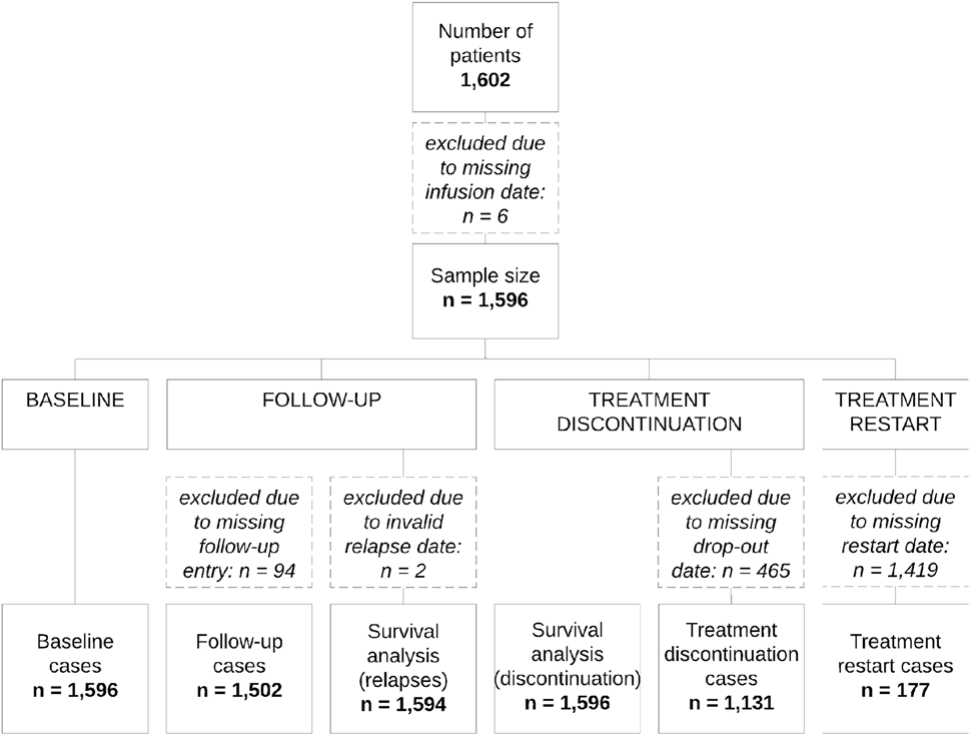
Inclusion flowchart. included patients as well as exclusions from the analysis at baseline, follow-up, treatment discontinuation and treatment restart with respective justification

Figure 1 gives an overview of the included and excluded patients, including the reasons for exclusion.

### Baseline

Table 1 describes the baseline characteristics of this cohort, which includes demographic data, clinical (ARR, EDSS) and paraclinical (MRI) markers of disease activity as well as prior DMT use.

**Table 1:** Baseline cohort characteristics

Baseline sample *n = *1596		
Demographics		
Female^b^	1133	71.0%
Age (years)^c^	33	13–67
Disease activity		
ARR (in the year before NTZ)^c^	2.0	0–8
EDSS^2^	2.5	0–8.5
≥ 9 lesions in T_2_-weighed MRI^b^	1466	92.6%
≥ 1 contrast-enhancing lesions^b^	1025	64.8%
Increased T_2_ lesion load^b^	1163	73.5%
Indication A^bd^	1172	73.4%
Indication B^ab^	403	25.3%
Time since diagnosis (months)^c^	67	0–473
Prior DMT		
Glatiramer acetate 20 mg	383	22.4%
Interferon β-1a i.m	372	21.7%
Interferon β-1a s.c. 44 µg	265	15.5%

*ARR* annualized relapse rate, *NTZ* natalizumab, *EDSS* expanded disability status scale, *MRI* magnetic resonance imaging, *DMT* disease modifying treatment

^a^ ≥ 2 severe relapses without preceding DMT and ≥ 1 gadolinium-enhancing lesion or increase in T_2_ lesion load as compared to a previous scan

^b^Absolute number and percentage

^b^Median and range

^d^ ≥ 1 relapse despite use of DMT within the past 12 months and ≥ 9 T_2_ lesions

### Follow up

The analysis of follow-up visits included 1502 patients who accounted for 20,101 follow-up entries. Patients had a median number of 12 (2–93) follow-up visits and a median time of 4.3 years until last follow-up.

### Efficacy

**Fig. 2:**
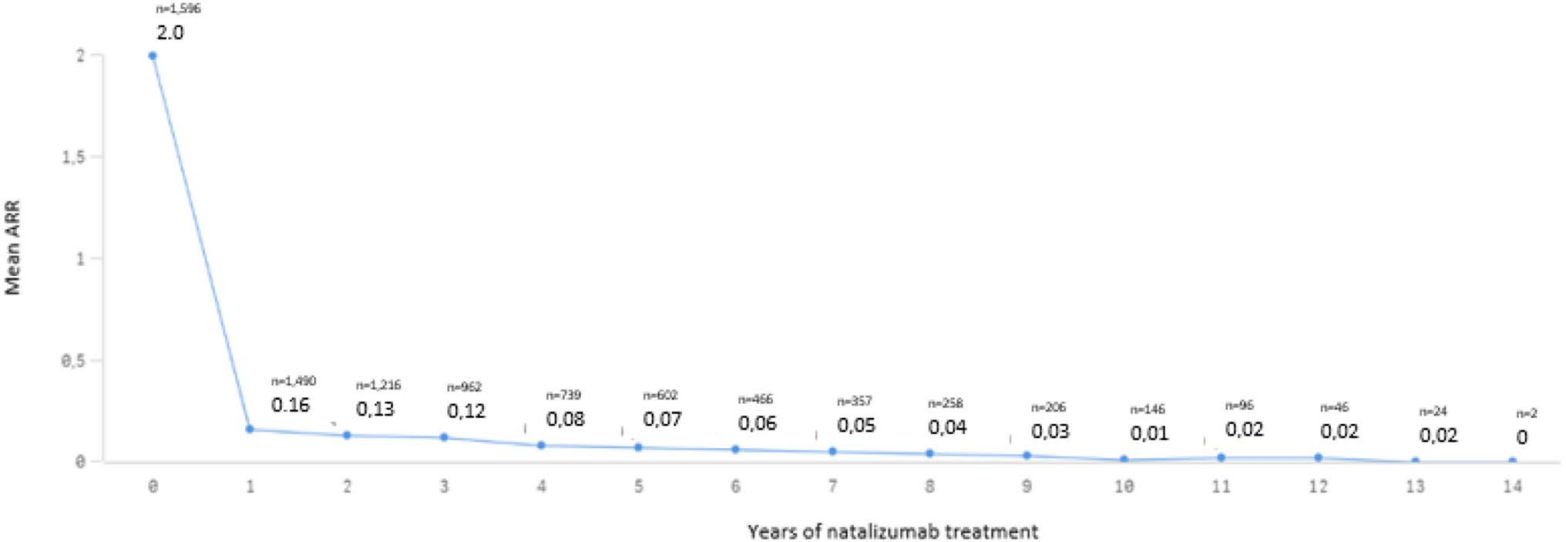
Mean ARR (= annualized relapse rate) across treatment intervals (interval 1 = 0–12 months, interval 2 = 13–24 months, interval 3 = 25–36 months, etc.) with corresponding case numbers

The efficacy of NTZ is illustrated in Fig. 2 with a mean ARR of 0.13 after 2 years and 0.01 after 10 years compared to a mean ARR of 2.0 at baseline.

A total of 432 relapses were documented during the observational period of 0–164 months in 1594 patients. The results indicated a mean survival time (time without relapse) of 112 months (standard error of the mean [SEM] = 2.14) respectively 9.3 years (75th percentile 31 months/2.6 years).

**Fig. 3:**
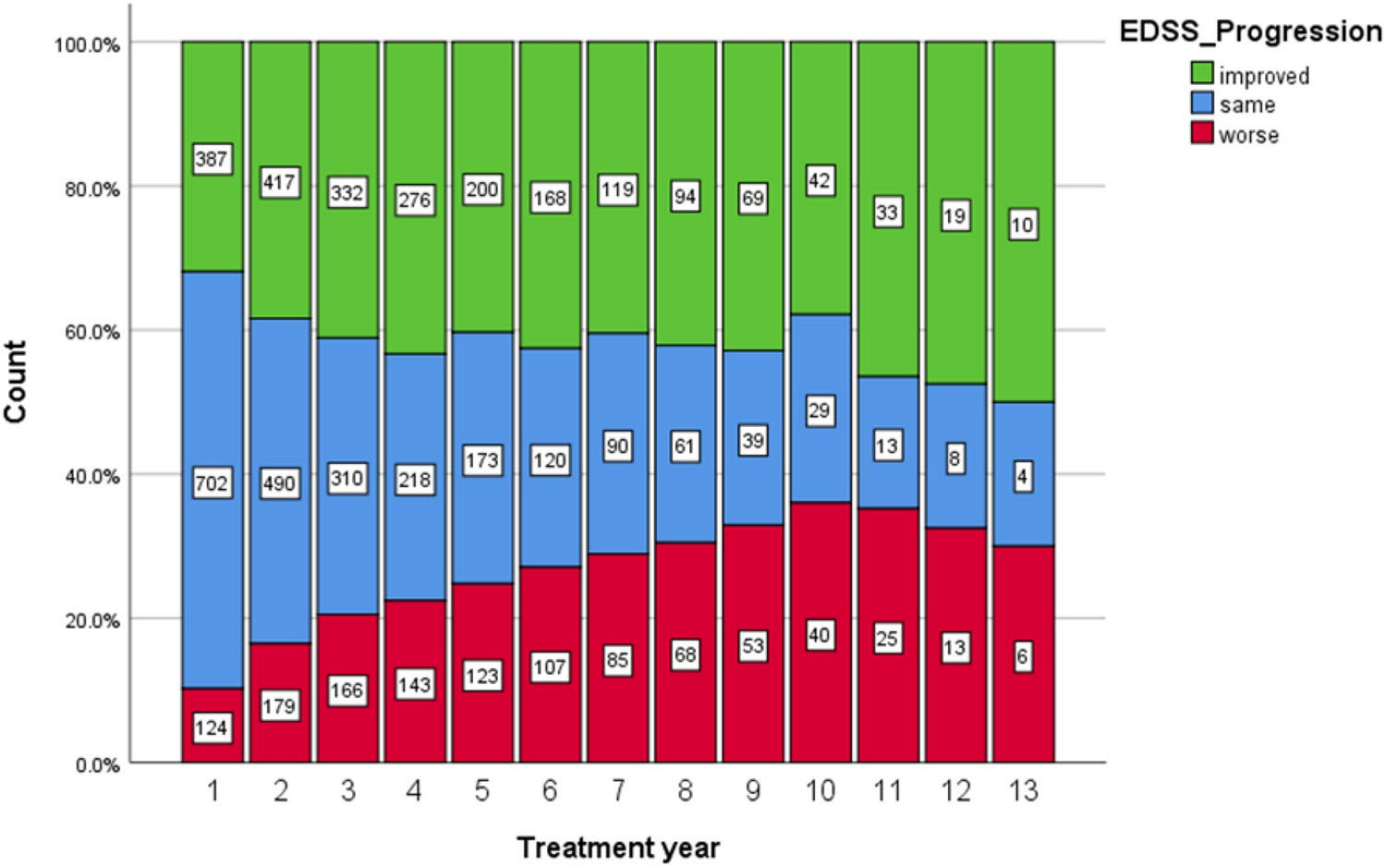
EDSS (= expanded disability status scale) Progression with treatment duration in years on the x-coordinate and the count of patients in percentages (absolute numbers within the bars) on the y-coordinate

A significantly higher EDSS score (3.26 vs 2.8; *p < *0.001) was shown for those with an on-treatment relapse. Baseline ARR values similarly reflected a significant group difference (2.3 vs 1.99 ARR; *p < *0.001). There was no difference between on-treatment relapse versus no on-treatment relapse regarding age or disease duration at baseline. A crosstab showed that 840 (52.7%) patients without on-treatment relapses met indication A at baseline and 332 (20.8%) patients met indication B. Among those who experienced an on-treatment relapse, 310 (19.5%) met indication type A and 93 (5.9%) indication type B at baseline (missing data for 1.1%). A *χ*^2^-test showed a significant association between baseline indication and on-treatment relapse (*χ*^2^ (1) = 4.196; *p = *0.041; *φ = *0.41). EDSS progression is illustrated in Fig. 3.

*N = *325 (21.6%) patients displayed EDSS worsening. For the proportion of patients converting to SPMS per treatment year, see Table 2.

**Table 2:** Proportion of patients converting to SPMS per treatment year

Treatment year	Frequency	Percent	Valid percent	Cumulative percent	Percent related to FU cohort^a^ (%)
1.00	33	10.2	10.2	10.2	2.2
2.00	53	16.3	16.3	26.5	4.36
3.00	44	13.5	13.5	40.0	4.57
4.00	34	10.5	10.5	50.5	4.60
5.00	45	13.8	13.8	64.3	7.48
6.00	28	8.6	8.6	72.9	6.00
7.00	20	6.2	6.2	79.1	5.60
8.00	27	8.3	8.3	87.4	10.4
9.00	20	6.2	6.2	93.5	9.70
10.00	8	2.5	2.5	96.0	5.48
11.00	12	3.7	3.7	99.7	0.13
12.00	1	.3	.3	100.0	2.17
Total	325	100.0	100.0		21.64

^a^last column shows percentages related to the Follow up (FU) cohort per respective treatment year see Table 2;

Patients who converted to SPMS within in the first 4 years were significantly older (median 49a vs 43a, *p < *0.001), more often male (*p < *0.001), had a higher EDSS score at baseline (median EDSS 3 vs 2, *p < *0.001), and more often met indication A as compared to indication B criteria(*p < *0.001). Noteworthy, data concerning disease duration and ARR did not differ at baseline.

### Safety

Out of the 1502 patients included in the analysis of follow-up visits, 1297 (86.4%) patients never indicated any Med DRA (Medical Dictionary for Regulatory Activities) code for adverse events (AE) and *n = *205 (13.6%) patients reported at least one Med DRA code. The Med DRA codes reported for most patients were (multiple reports possible): code 1; infections and infestations (*n = *51; 24.8%), code 22; general disorders and administration site conditions (*n = *48; 23.4%) and code 8; nervous system disorders (*n = *45; 21.9%). A detailed overview is given in Table 3.

**Table 3:** Med DRA (= Medical Dictionary for Regulatory Activities) codes across follow-up visits (number of Med DRA Code reports, in some cases several reports were made for one patient)

MedDRA SOC codes	*n*	%
Infections and infestations	51	17.5
General disorders and administration site conditions	48	16.4
Nervous system disorders	45	15.4
Blood and lymphatic system disorders	22	7.5
Skin and subcutaneous tissue disorders	21	7.2
Gastrointestinal disorders	18	6.2
Respiratory, thoracic and mediastinal disorders	12	4.1
Vascular disorders	10	3.4
Musculoskeletal and connective tissue disorders	10	3.4
Examinations	10	3.4
Psychiatric disorders	8	2.7
Immune system disorders	6	2.1
Metabolism and nutrition disorders	6	2.1
Eye disorders	4	1.4
Injury, poisoning and procedural complications	4	1.4
Surgical and medical procedures	4	1.4
Renal and urinary disorders	3	1.0
Neoplasms benign, malignant and unspecified (including cysts and polyps)	2	0.7
Ear and labyrinth disorders	2	0.7
Cardiac disorders	2	0.7
Reproductive system and breast disorders	2	0.7
Liver and gallbladder disorders	1	0.3
Social circumstances	1	0.3
Total	292	100.0

Cases of PML were documented separately. A total of 5 cases of PML were reported to the registry, of which 1 patient died of PML.

STRATIFY test for anti-JCV-antibodies had been reported for 1,100 (73.2%) patients with 618 (41.1%) patients testing positive at least once. *N = *73 (11.8%) of these had a negative test result at baseline and are therefore, likely to have undergone JCV seroconversion. In contrast, 135 (21.8%) already tested positive at baseline and the remaining 410 (66.3%) had no valid data entry for their baseline STRATIFY result.

*N = *714 (47.5%) patients were tested for neutralizing anti-NTZ-antibodies at least once, with 28 (1.9%) yielding a positive test result.

### Treatment discontinuation

Treatment discontinuation was documented for 1131 patients. Reasons for discontinuing NTZ treatment are summarized in Table 4, with JCV seropositivity (including a few cases with fear of PML instead of JCV seropositivity as specification) being by far the most frequent reason (*n = *607, 53.7%).

**Table 4:** Reasons for discontinuing natalizumab treatment

Discontinuation (*n = *1131)	*n*	%
Treatment discontinuation	1131	100%
JCV positivity/fear of PML	607	53.7%
Disease course	146	12.9%
EDSS progression only	(43	29.5%)
Stable	(39	26.7%)
MRI progression only	(23	15.8%)
Relapse	(21	14.4%)
EDSS and MRI progression	(20	13.7%)
Pregnancy/planned pregnancy	108	9.5%
Patent´s wish without specified reason	92	8.1%
Side effects	54	4.8%
Infusion reaction/allergy	(14	26%)
Infections	(12	22.3%
PML	(5	9.3%)
Malignancy	(5	9.3%)
Aggravation of MS-Symptoms (infusion related)	(5	9.3%)
Liver function parameters elevated	(4	7.4%)
Herpes zoster infection	(2	3.7%)
Anaemia	(2	3.7%)
Headache (infusion related)	(1	1.8%)
Diabetes mellitus: hyperglycaemia with every infusion	(1	1.8%)
Perimyocarditis	(1	1.8%)
Interstitial lung disease	(1	1.8%)
Bipolar-affective disorder	(1	1.8%)
No specified reason	54	4.8%
Natalizumab neutralising antibodies	25	2.2%
Malcompliancy (doctor´s decision)	19	1.7%
Other reasons (see table description)	11	1%
Fear of therapy (excluding PML)	9	< 1%
Frustral peripheral vein situation	6	< 1%

JCV John Cunningham virus, *PML* progressive multifocal leukoencephalopathy, *EDSS* expanded disability status scale, *MRI* magnetic resonance imaging; other reasons included 5 patients without cost coverage, 4 due to study inclusion, 1 with death due to an accident and 1 due to kidney transplantation;

A total of 714 (63.1%) of cases reported that a follow-up treatment after NTZ discontinuation was planned. Fingolimod was the most frequently reported follow-up therapy (377; 52.8%), followed by glatiramer acetate (63; 8.8%) and intravenous immunoglobulins (IvIg) therapy (37; 5.2%). “Not specified” was indicated for 82 (11.6%) patients.

**Fig. 4:**
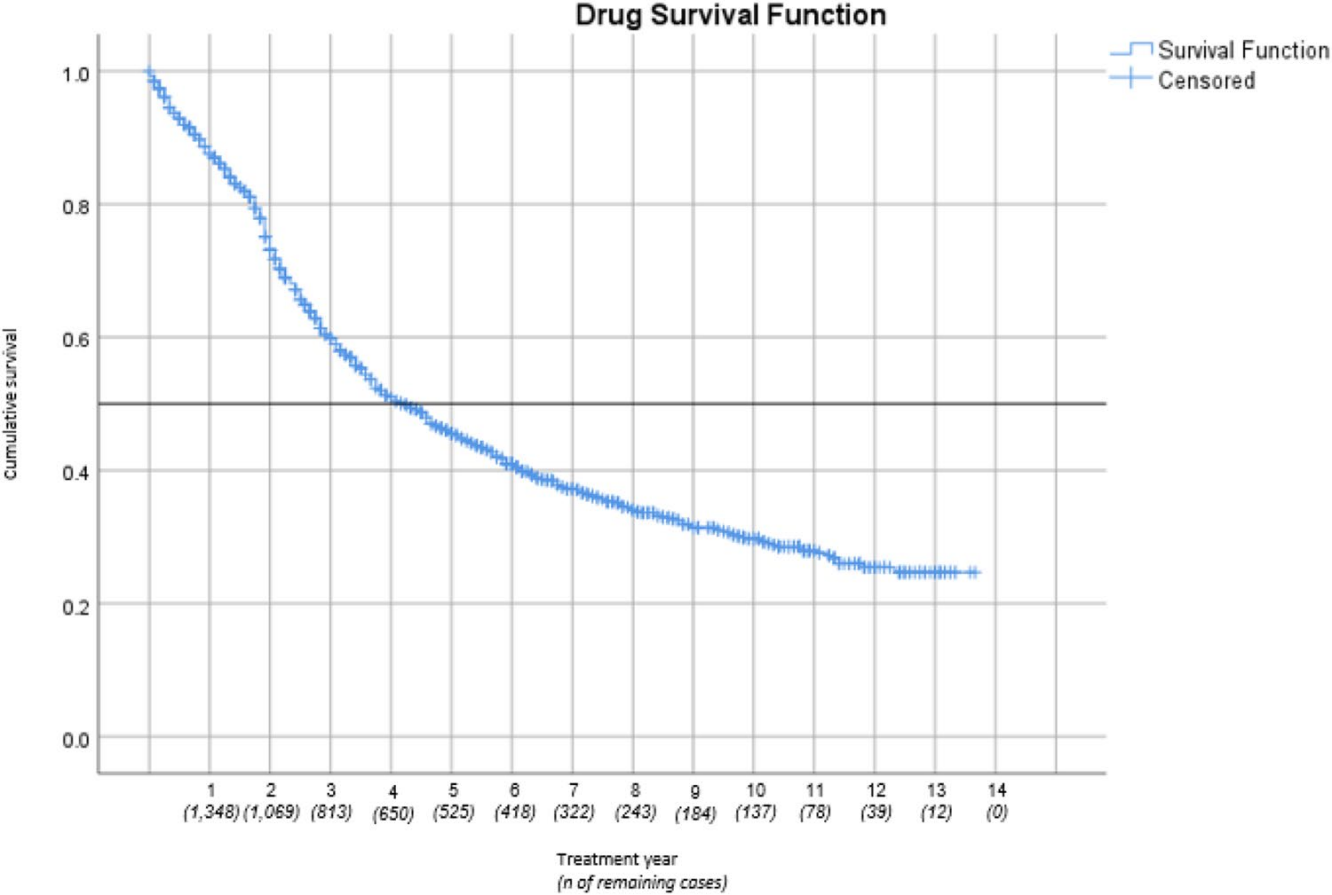
Kaplan Meier curve concerning drug survival

A Kaplan–Meier curve was created to illustrate drug survival (Fig. 4). Results showed a median survival time of 51 months (4.3 years) with the first percentile at 148 months (12.3 years) and the 75th percentile at 24 months.

### NTZ treatment restart

*N = *177 patients accounting for 313 data entries were registered to have restarted NTZ treatment. The median duration of treatment pause was 11 months with a range from 0 to 125 months. EDSS scores at restart showed a median of 2.5 and ranged from 0.0 to 7.5. Reoccurrence of relapses was reported as a cause for NTZ restart in 34.5% of cases with a majority (*n = *42; 23.7%) indicating that only one relapse occurred. Two relapses were reported in 13 (7.3%) cases, three relapses were reported in 4 (2.3%) cases and 2 (1.1%) cases had five relapses.

## Discussion

With up to 14 years of follow-up, the AMSTR provides critical insights into the effectiveness and safety profile of long-term NTZ use in a real-world setting.

Here we provide data confirming the effectiveness of NTZ in a real-world cohort of highly active RRMS patients. The ARR was reduced from 2.0 at baseline to 0.16 (92% reduction of ARR compared to baseline) in the first year of treatment and further decreased to 0.01 (> 99% reduction of ARR compared to baseline) after 10 years.

Moreover, patients remained relapse-free for a mean of 9.3 years, and the proportion of no on treatment relapse was significantly (*p = *0.041) higher for those with indication type B (*early intensive strategy)* reinforcing the meaningfulness of an early active treatment strategy[10]. This is consistent with real-world data available so far and underlines the established use of NTZ as first-line therapy for highly active RRMS patients [7, 11–13].

Conversion to secondary progressive MS (SPMS) was observed in 21.6% with a median age of 43 years, representing a typical age of conversion to SPMS [8]. Secondly, with a median disease duration of 14 years, patients were showing a relatively low conversion rate with a relatively late time point of conversion compared to natural history data, especially when considering the fact that this cohort consists of patients with highly active RRMS [14]. Indeed, according to natural history data, a conversion rate to SPMS of around 50% would be expected within 19 years in a cohort with mild to highly active disease courses [15, 16].

Similar to the effectiveness data, our long-term safety results are also in line with available real-world data. The vast majority of the patients (86.4%) did not report any AE. The most common reported AEs were (i) infections and infestations, (ii) general disorders and administration site conditions, and (iii) nervous system disorders.

This is consistent with the findings in the pivotal phase III studies (AFFIRM, SENTINEL), where a slightly increased incidence of urinary tract infections and respiratory infections was observed [5, 6].

In the AMSTR a total of 5 PML cases among 1596 patients (5 confirmed PML cases with 1 death), were captured. Compared to other large surveillance programs (TOP, TYGRIS, STRATA, STRATIFY-2, see Table 5), our data revealed comparable AE rates including PML cases [7, 11–13].

**Table 5:** Comparison of major natalizumab observational studies (TOP, TYGRIS, STRATA, STRATIFY-2) as well as the AMSTR cohort

	TOP (*n = *6148)	TYGRIS (*n = *6508)	STRATA (*n = *1094)	STRATIFY-2 (*n = *24,402)	AMSTR (*n = *1596)
Females	*n = *4430 (72.1)	*n = *4749 (73)	*n = *755 (69)	17,938 (74)	*n = *1133 (71)
Age at baseline (years)	37.1 (30–44)	40.1 (10·4)	41.4 (35–48)	44.1 (36–52)	33 (SD)
Disease duration (baseline)	7.8 (0–48)	9.6 (7.3)	8 (4–34)	n.d	5.6 (0–39)
EDSS (baseline)	3.5 (1.6)	n.d	2.5 (0–8)	n.d	2.5 (0–8.5)
ARR (baseline)	2.0 (1.0)	n.d	1 (0–8)	n.d	2 (0–8)
SAE ≥ 1	829 (13.5%)	15.3%	16%	n.d	13.6%
infections	254 (4.1%)	212 (3.3%)	44 (4%)	n.d	3.4%
PML (confirmed)	53 (0.9%)	44 (0.7%)	8 (0.7%)	65 (0.26%)	5 (0.3%)

*EDSS*  Expanded Disability Status Scale, *ARR* annualised relapse rate, *SAE* severe adverse event, *PML* progressive multifocal leukoencephalopathy, *n.d.* no data available, median/mean values given with standard deviation or 95% confidence interval (CI)

Hereafter, Table 5 provides a comparison of the different observation programs with the AMSTR data [7, 11–13].

Certain differences could be explained by different sample sizes and differences in data reporting and documentation, i.e., the AMSTR represents a nationwide registry, covering most patients treated with NTZ in Austria, while TOP is an open-label, multinational observational study. Furthermore, it could be due to the smaller sample size in comparison with the international registry studies (see Table 5).

All in all, NTZ represents a clear first-line therapy in the treatment of (highly) active RRMS with an excellent safety profile. The only limiting factor is seroconversion of JCV, especially after 2 or more years of therapy.

The main potential limitations of this registry-based study are: (i) adverse events may not have been reported to the registry (reporting bias), (ii) the number of patients decreased significantly over years on treatment (attrition bias), given a caseload of less than 100 after year 10. Hence, we must emphasize that this impressing efficacy data need to be interpretated with caution as the relatively high drop-out rates in our study may lead to a selection bias towards stable disease patients continuing the study.

## Conclusion

In line with real world data available so far, we confirm in a nationwide Austrian registry study the high effectiveness as well as the long-term safety of NTZ in highly active RRMS patients. In particular, no new safety issues including PML numbers occurred, which is in line with international surveillance programmes [7, 11–13]. Our data, therefore, support the use of NTZ as a first line therapy in active RRMS patients due to its excellent benefit-risk-profile, with JCV-seroconversion being the only major limitation in the long-term use of NTZ.

## Data Availability

Anonymized data will be shared by reasonable request from any qualified investigator.
