# Correction: Real-world use of natalizumab in Austria: data from the Austrian Multiple Sclerosis Treatment Registry (AMSTR)

**DOI:** 10.1007/s00415-023-11784-1

**Published:** 2023-06-05

**Authors:** Tobias Monschein, Sarinah Dekany, Tobias Zrzavy, Markus Ponleitner, Patrick Altmann, Gabriel Bsteh, Barbara Kornek, Paulus Rommer, Christian Enzinger, Franziska Di Pauli, Jörg Kraus, Thomas Berger, Fritz Leutmezer, Michael Guger, the Austrian MS Treatment Registry (AMSTR)

**Affiliations:** 1 Department of Neurology, Medical University of Vienna, Waehringer Guertel 18-20, 1090 Vienna, Austria; 2 Department of Neurology, Medical University of Graz, Graz, Austria; 3 Department of Neurology, Medical University of Innsbruck, Innsbruck, Austria; 4 Department of Neurology, Medical Faculty, Heinrich Heine University Düsseldorf, Düsseldorf, Germany; 5 Department of Laboratory Medicine, Paracelsus Medical University and Salzburger Landeskliniken, Salzburg, Austria; 6 Clinic for Neurology 2, Kepler University Clinic, Linz, Austria

**Correction: Journal of Neurology** 10.1007/s00415-023-11686-2

The original version of this article unfortunately contained a mistake. Affiliation detail for Author Christian Enzinger was incorrectly given as

^2^Department of Neurology, Karl-Franzens University, Graz, Austria

but should have been

^2^Department of Neurology, Medical University of Graz, Graz, Austria

